# Survival Analysis in Patients with Laryngeal Cancer: A Retrospective Cohort Study

**DOI:** 10.3390/life13020295

**Published:** 2023-01-20

**Authors:** Elena Molina-Fernández, José M. Palacios-García, Ramón Moreno-Luna, Tomás Herrero-Salado, Julio Ventura-Díaz, Serafín Sánchez-Gómez, Ángel Vilches-Arenas

**Affiliations:** 1 Department of Otorhinolaryngology and Head and Neck Surgery, University Hospital Virgen Macarena, Doctor Fedriani 3, 41009 Seville, Spain; 2 Department of Preventive Medicine and Public Health, University Hospital Virgen Macarena, Doctor Fedriani 3, 41009 Seville, Spain

**Keywords:** laryngeal cancer, organ preservation, laryngectomy, survival

## Abstract

Introduction: The worldwide incidence rate of laryngeal cancer is declining. However, the 5-year survival for these patients has decreased in recent years from 66% to 63%. This may be due to changes in the treatment of the disease. The present study aimed to evaluate the survival rate of patients with LC according to the stage of the disease and the treatment applied. For this purpose, surgical versus organ preservation protocols (OPP) based on chemoradiotherapy were evaluated. Methods: A retrospective cohort study was conducted in a tertiary hospital. The study included adult patients with a clinical diagnosis of primary LC. Patients with LC and systemic metastases and those with synchronous tumors at diagnosis were excluded. Univariate and multivariate analyses were performed to determine the association between exposure to LC treatment and the time to event (death). Overall survival (OS), cause-specific survival (CSS), and disease-free survival (DFS) were calculated. Results: Patients with advanced tumors (stages III and IV) had almost three times the risk of LC death than those in the initial tumor stages (I and II) [HR CCS = 2.89 (95%CI 1.30–6.39)]; [HR OS = 2.01 (95%CI 1.35–2.98)]. Patients who underwent surgical treatment had a higher chance of survival than those who were treated according to OPP [HR = 0.62; 95%CI (0.38–1.02)] in CSS, 0.74 [95%CI (0.50–1.90)] in OS, and 0.61 [95%CI (0.40–0.91)] in DFS. Discussion: OPP changed the management of patients with advanced stages of LC, establishing CRT as an alternative to surgery. Our data did not reveal clinically relevant differences in OS between patients treated with OPP and those who underwent surgery; however, we reported differences in the DFS rate after five years of follow-up in favor of the surgery-treated group of patients. Conclusion: Surgical treatment improves CSS and DFS at five years in patients with initial LC with respect to radiation therapy alone. Furthermore, surgical treatment associated with complementary radiation therapy offers better CSS and DFS in patients with advanced LC.

## 1. Introduction

The World Health Organization estimates that laryngeal cancer (LC) accounts for more than 180,000 new annual cases worldwide, and more than 100,000 of the cases end up in death [1]. Tobacco use represents the main risk factor for the development of LC. Active smokers are at seven times higher risk of developing LC than nonsmokers [2]. The risk of occurrence of LC is present in active smokers and in people chronically exposed to tobacco smoke [3,4]. Alcohol consumption is the second-most important risk factor for LC [5,6]. Co-exposure to tobacco and alcohol produces a synergistic effect that increases the risk of developing LC [6,7].

LC management has evolved over the years. Traditionally, radical surgical treatment with total laryngectomy was the treatment of choice even in the early stages of the disease. Laryngeal preservation surgeries were then developed to preserve the voice in the early stages of LC [8]. The introduction of organ preservation protocols (OPP) using chemoradiotherapy treatment by the Department of Veterans Affairs group in 1991 [9] significantly changed the management of LC. Currently, LC treatments are classified as initial (I and II) and advanced (III and IV) stages of the disease according to the TNM (tumor, node, and metastasis) classification of the American Joint Committee on Cancer (AJCC) and the Union Internationale Contre le Cancer (UICC) [10]. Early-stage LC patients can now be treated with a single treatment modality [11], surgical or radiotherapeutic. Both therapeutic options produce very similar results in terms of the local control and survival [12,13,14]. The 5-year disease-free survival rates for stage I and stage II LC are 90% and 80%, respectively [15]. Advanced LC is generally treated using any of the following options: a combination of surgery followed by radiotherapy or chemoradiotherapy, concomitant chemoradiotherapy, or induction chemotherapy followed by radiotherapy or surgery, depending on the tumor response to induction [16,17]. Selected stage III LC patients who undergo organ preservation surgery and with signs of a good prognosis can be treated with surgery alone [18].

Despite the recent advances mentioned above in LC management and the decrease in disease incidence, the 5-year survival rate of LC patients has decreased from 66% to 63% [19]. This decrease in survival could be due to the change in the treatment paradigm in patients with advanced stages of LC with the introduction of OPP, which decreases surgical therapy. Recent studies such as those of Megwalu et al. [20] and Wolf et al. [21] have shown that the OS and DSS rates are higher in patients undergoing surgical treatment versus OPP.

Therefore, the present study aimed to evaluate the survival rate of patients with LC according to the stage of the disease and the applied treatment. To this end, we evaluated surgical versus OPP treatment modalities based on chemoradiotherapy.

## 2. Methods

### 2.1. Study Design and Settings

A retrospective cohort study was conducted in a tertiary hospital of the University Hospital Virgen Macarena, Seville, Spain, between 2006 and 2017. The study included adult patients (>18 y) with a clinical diagnosis of primary LC. Patients with LC and systemic metastases and those with synchronous tumors at diagnosis were excluded. 

### 2.2. Ethics

All patients signed an informed consent prior to the procedure, and the data collection was anonymized. This study was approved by the Ethics Committee of the Virgen Macarena and Virgen del Rocio University Hospitals (protocol number: 0674-N-18).

### 2.3. Data Collection

Data on the following variable were recovered from medical records of patients: sociodemographic variables (sex and age); habits such as smoking and alcohol consumption; exposure to parenteral drugs; tumor details including stage (T and N stages), location and anatomopathological result; received tumor therapy (initial treatment performed, type of surgery, administration of chemotherapy, and complementary radiotherapy) and complications arising from LC treatment (pharyngeal fistula and performance of reconstructive flaps); and survival variables (overall survival (OS), cause-specific survival (CSS), disease-free survival (DFS), local control, and regional control).

The definition of exposure was for patients undergoing laryngeal surgery (TLM, partial resection, subtotal resection, or total resection) as the main treatment versus those undergoing OPP (radiotherapy alone or chemoradiotherapy). 

### 2.4. Statistical Analysis

Data were analyzed using the IBM Corp. statistical package released in 2013: IBM SPSS Statistics for Windows, Version 22.0, Armonk, NY, USA. Categorical variables were expressed as frequencies and percentages. Quantitative variables were reported as the arithmetic mean (x) and standard deviation (SD) if the data followed a normal distribution; otherwise, they were summarized as the median (Me) and interquartile range (IQR). The Kolmogorov–Smirnov test was applied to verify the normal distribution of the variables. 

A univariate survival analysis was performed using a Kaplan–Meier curve, where the independent variable was the type of treatment. The log-rank test was used to contrast the hypothesis of equality of the survival time distributions between the different groups of treatment under study. 

Univariate and multivariate analyses were performed using Cox regression models to determine the association between exposure to LC treatment and time to event (death). Hazard ratios (HR) and their 95% confidence intervals (CI) were estimated. A *p*-value of 0.15 was established for the univariate analysis, while, for the multivariate analysis, a *p*-value < 0.05 was considered statistically significant.

In all hypothesis tests, a significance level <0.05 was considered statistically significant.

## 3. Results

Three hundred and sixteen patients met the inclusion criteria and were included in the study. The mean age of the participants was 62.6 ± 11.1 years (Range: 24–91 years). Table 1 shows the baseline characteristics of the study population categorized according to the initial or advanced stages. 

The univariate analysis of the association of the risk factors under study with cause-specific survival (CSS), overall survival (OS), and disease-free survival (DFS) is summarized in Table 2. Patients with advanced stages (stages III and IV) had almost three times the risk of LC death than those in the initial tumor stages (I and II) [HR CCS = 2.89 (95%CI 1.30–6.39)]; [HR OS = 2.01 (95%CI 1.35–2.98)]. Being older than 70 years was associated with a substantially higher risk of LC death than patients 70 years or younger [HR CCS = 1.81 (95%CI 1.07–3.05)]. Alcohol consumption was also associated with an increased risk of death from LC and an increased risk of recurrence [HR CCS = 2.03 (95%CI 1.24–3.33)]; [HR DFS = 1.61 (95%CI 1.10–2.37)].

Patients with advanced stages T (T3–T4), advanced stages N (N2–N3), and advanced stages of TNM (III–IV) had a higher risk of death from CSS and OS than patients with the initial stages (Table 2). 

Patients who underwent surgical treatment had a higher survival chance than those who were treated according to OPP [HR = 0.62 (95%CI 0.38–1.02)] in CSS, 0.74 (95%CI 0.50–1.90) in OS, and 0.61 (95%CI 0.40–0.91) in DFS (Table 2). Postoperative radiation therapy (PORT) is associated with a decreased risk of developing recurrences [HR = 0.46 (95%CI (0.26–0.80)]. The preservation of laryngeal functionality after radiation therapy (RT) administration is associated with an 89% increased probability of cause-specific survival after LC [HR = 0.11 (95%CI 0.05–0.28)] and with an overall survival rate of 63% [HR = 0.37 (95%CI 0.20–0.66)]. The development of local, regional recurrence and the administration of chemotherapy (CHT) were associated with an increased risk of cause-specific death, as well as general death (Table 2).

The multivariate analysis of CSS showed that, after controlling for TNM staging, surgical treatment is associated with a lower risk of death [HR = 0.58 (95%CI: 0.35; 0.98)] as compared to OPP (Table 3). For OS, regardless of the stage of LC, being older than 70 years, regional recurrence of LC, and the administration of salvage QT were associated with an increased risk of death [HR = 4.23 (95%CI 2.14–8.34)], [HR = 3.48 (95%CI 1.62–7.50)], and [HR = 3.09 (95%CI 1.28–7.98)], respectively. In contrast, the preservation of laryngeal functionality after RT was associated with a 55% increased chance of survival [HR = 0.45 (95%CI 0.23–0.87)]. Regarding DFS, being more than 70 years of age and consuming alcohol were associated with an increased risk of developing recurrences [HR= 1.81 (95%CI 1.13–2.90)] and HR= 1.77 (95%CI 1.20–2.63)], respectively. On the contrary, PORT administration was associated with a 56% reduction in the risk of recurrence of LC [HR = 0.44 (95%CI 0.25–0.78)] (Table 3).

**Figure 1:**
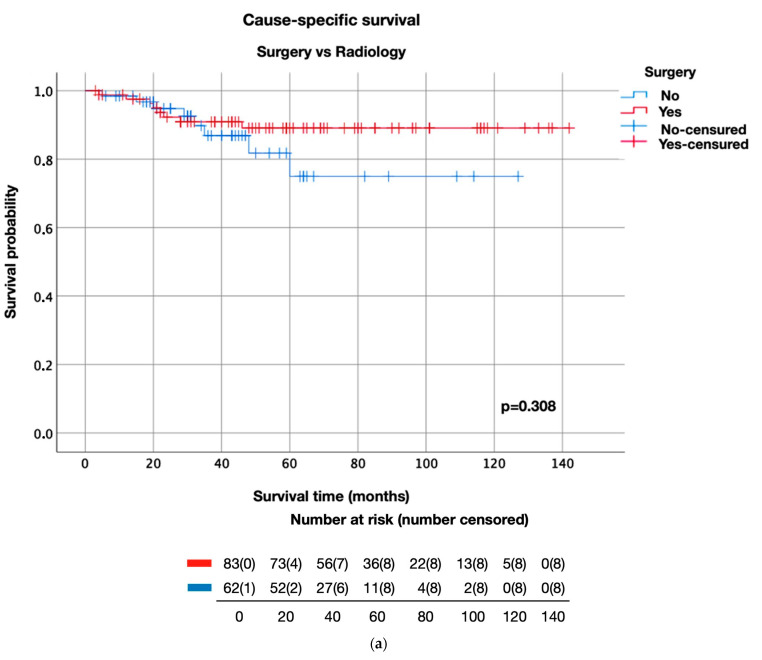

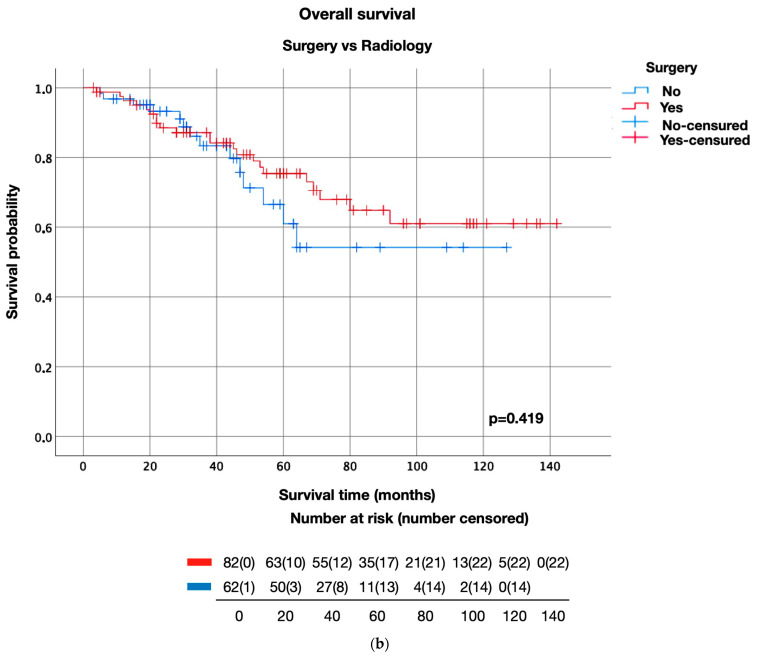

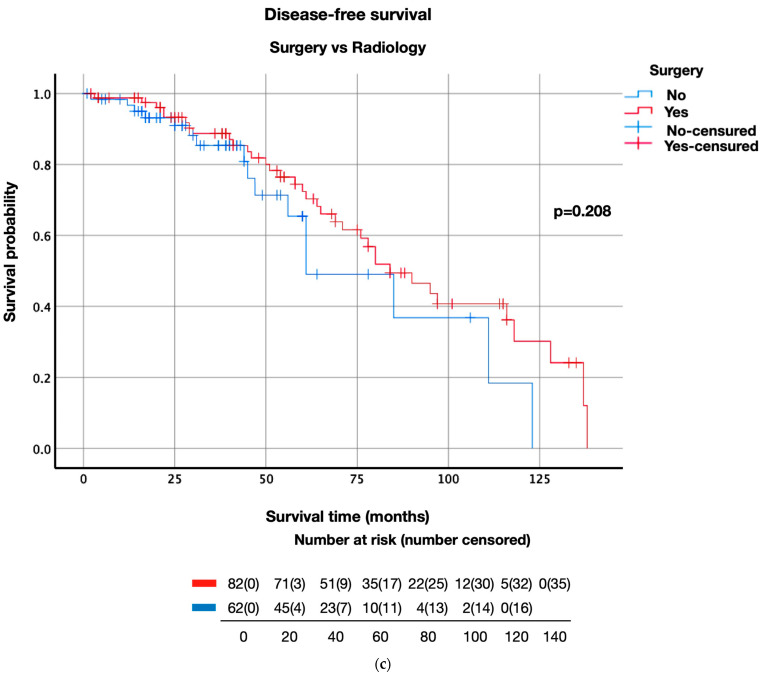
Survival in the early stages according to treatment. (**a**) CSS of early stages according to treatment. (**b**) OS in early stages according to treatment. (**c**) DFS of the early stages according to treatment.

**Figure 2:**
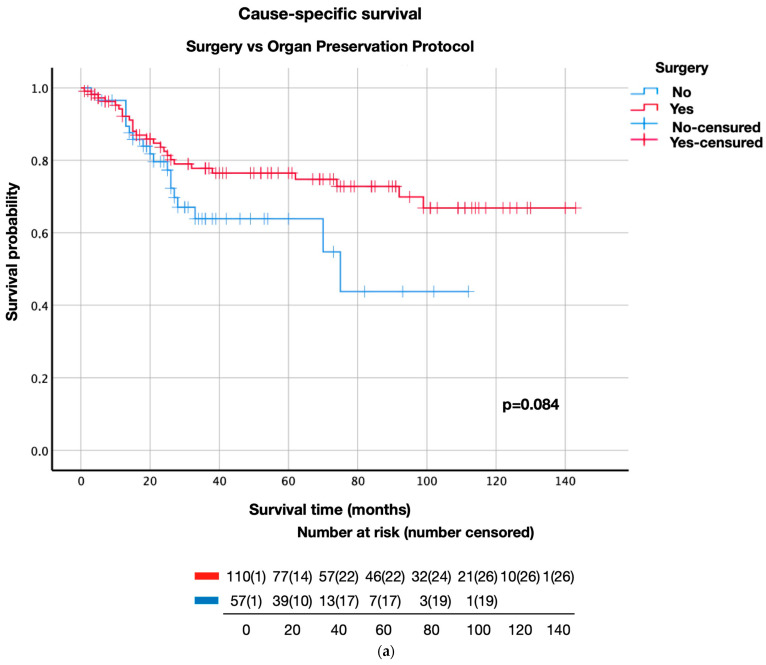

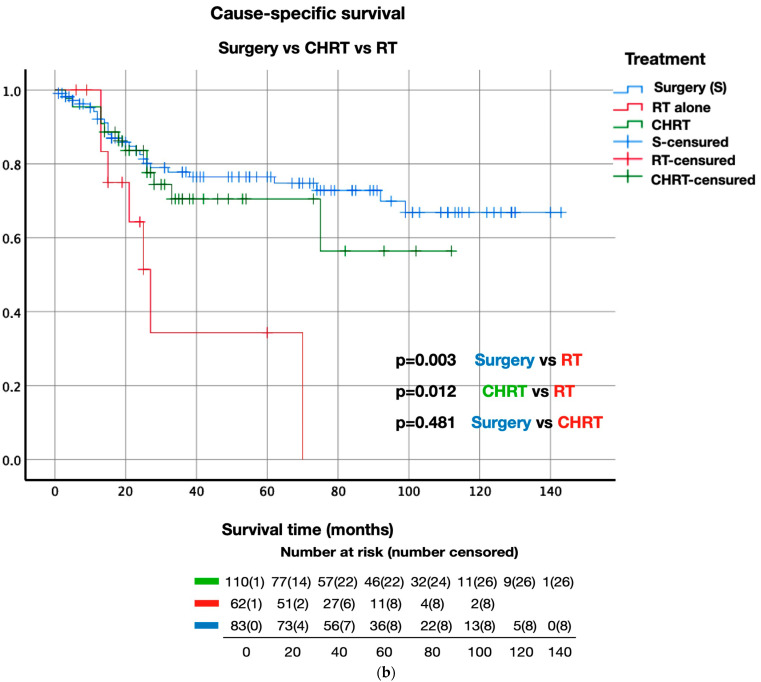

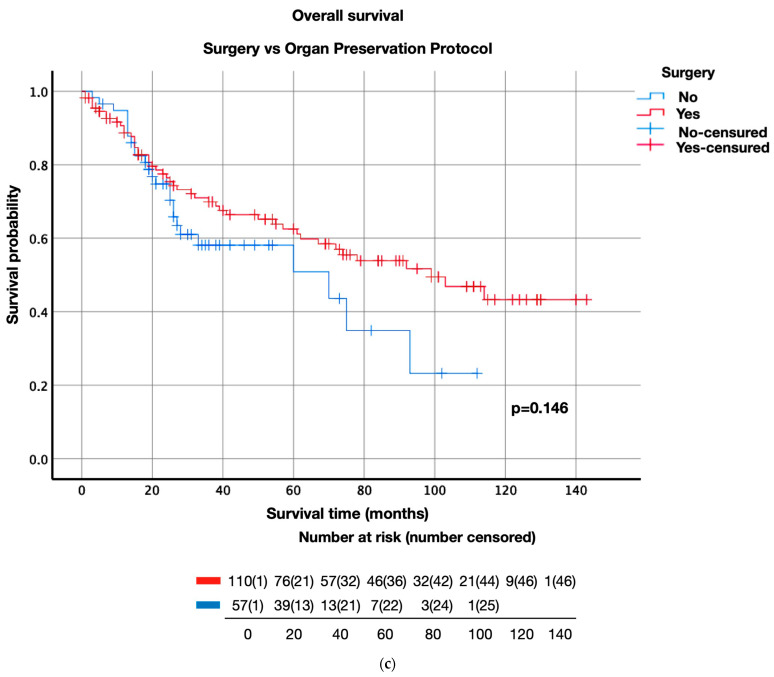

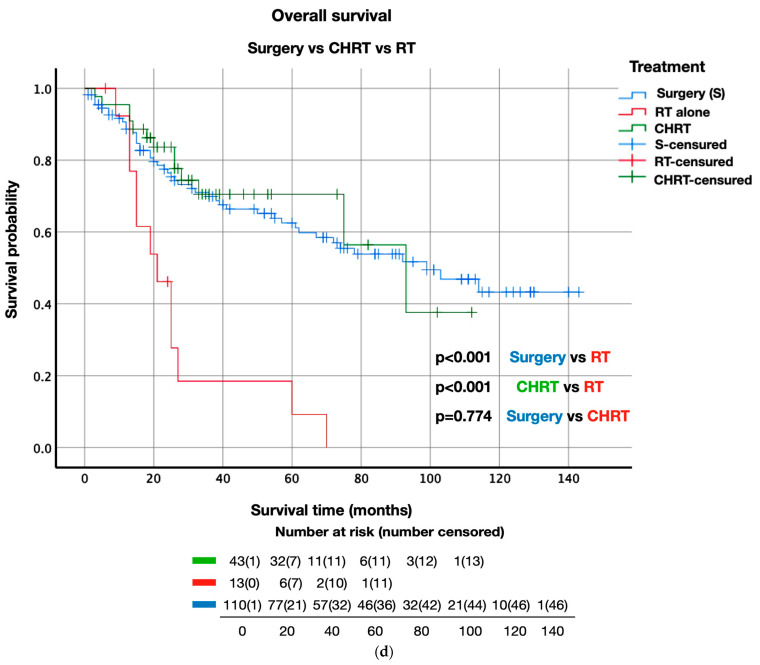

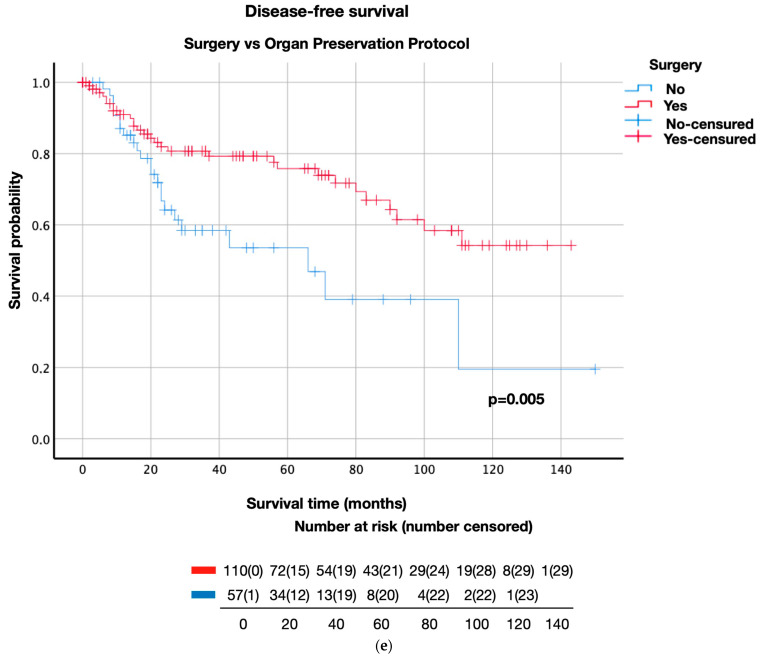

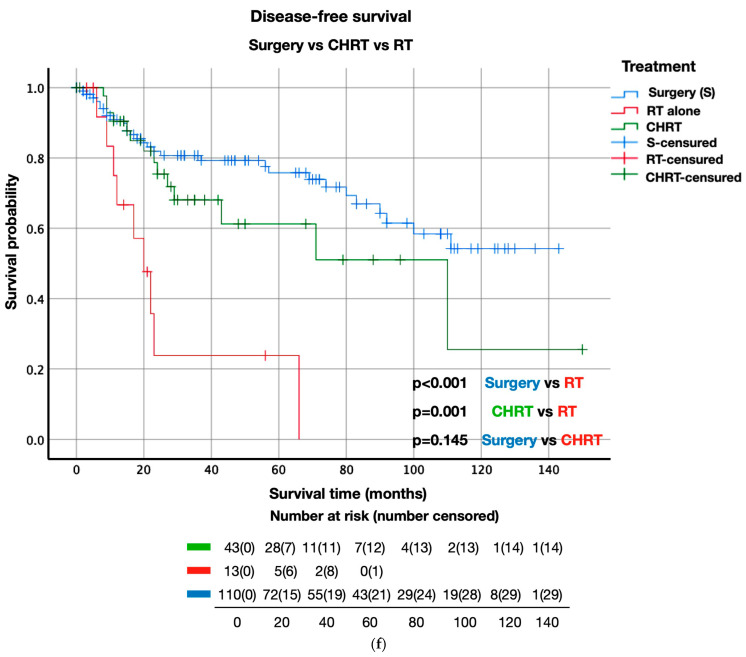
Survival of advanced stages according to treatment. (**a**) CSS of advanced stages according to surgery versus OPP. (**b**) CSS of advanced stages according to surgery versus CHRT versus RT. (**c**) Advanced-stage OS of advanced stages according to surgery versus OPP. (**d**) OS of advanced stages according to surgery versus CHRT versus RT. (**e**) DFS of advanced stages according to surgery versus OPP. (**f**) DFS of advanced stages according to surgery versus CHRT versus RT.

The survival curve analysis for CSS, OS, and DFS was performed by comparing the results obtained after surgery with those obtained after radical radiotherapy in the initial stages of LC and surgery versus OPP globally and independently for the two therapeutic strategies included in this group (chemoradiotherapy and radical radiotherapy) (Figure 1 and Figure 2). In the early stages of LC, no significant differences (*p*-value = 0.419) were observed between the impact of surgery and that of RT on CSS; however, after 5 years of follow-up, the chance of survival was substantially higher in the surgery group (89.1%) than in the radiation therapy group (75%) (Figure 1). Concerning DFS, clinically relevant differences were found after 5 years of follow-up with a survival rate of 72.4% in the surgery group and 49% in the RT group. These differences were maintained at 10 years of follow-up (Figure 1). In the advanced stages of LC (Figure 2), no statistically significant differences were observed in DFS (*p* = 0.084), OS (*p* = 0.156), and CSS (*p* = 0.009). However, the CSS analysis revealed clinically superior results during the entire follow-up of surgery patients versus OPP patients; the CSS of LC patients increased from 54.7% in the OPP group to 74.8% in the surgery group after five years of follow-up. Similarly, after 2 years of follow-up, the DFS patients increased from 65.9% in the OPP group to 81.9% in the surgery group (Figure 2). However, no significant differences in OS were observed in the patients who underwent surgery or OPP.

Independent analyses of the three treatments (surgery, radiation therapy, and chemoradiation (CRT)) showed a decrease in the estimated survival rates (CSS, OS, and DFS) in the group of patients who received radical intention radiotherapy compared to those who underwent surgery and in those who received CRT (Figure 2). Higher DFS estimates were obtained for patients who underwent surgery than for those who received CRT. These differences were more pronounced after 5 years of follow-up, although the differences were not statistically significant (Figure 2).

Stratifying the patients according to tumor stages (III and IV) to compare the survival rates between those patients who underwent surgery and those who received CRT with radical intent did not show any statistically significant differences between the two groups with respect to any of the survival rates (CSS, OS, and DFS) (*p* > 0.005). However, the likelihood of survival in the RT-treated group was less than in the other groups who received alternative treatments in stages III and IV of the disease (*p* < 0.005), except for patients with stage III LC, where the difference between survival rates after RT and CHRT treatments was not statistically significant (*p* = 0.070).

## 4. Discussion

The treatment of laryngeal cancer should be directed and individualized for the optimal results [22]. Patient factors such as comorbidities, nutritional status, and desires and tumor factors such as histological characteristics or tumor staging must be considered to propose the best treatment modality for each case. Our study shows the importance surgery continues to have for the treatment of laryngeal carcinoma. This is accurate for both early and advanced stages. However, OPP continues to play a role in certain cases where the patient’s pretreatment laryngeal function is good [23]. In this way, in the early stages of LC surgery, it seems to present higher chances of OS compared to radiotherapeutic treatment [24,25]. In our study, we did not find a significant increase in the OS rates, although we observed an increase of more than 10% in the long-term survival (five years of follow-up) of patients who underwent surgery compared to those who were treated with radiation therapy; perhaps with a longer follow-up duration, the observed difference in OS might reach statistical significance. Regarding CCS and DFS, we found clinically relevant differences in favor of surgical treatment after five years of follow-up. De Santis et al. [26] did not find these differences in 5-year disease-specific survival between the RT and surgery treatment groups. To the best of our knowledge, this study is the first to report such differences. Furthermore, surgery was postulated as a protective factor with a 42% lower probability of dying from LC [HR 0.58 (95%CI 0.35–0.98)] *p* = 0.042 and a 39% lower probability of recurrence of LC at any time during follow-up [HR 0.61 (95%CI 0.40–0.91)] *p* = 0.016.

The Veterans Study Group [9] introduced a change in the treatment of patients with advanced stages of LC, establishing CRT as an alternative to surgery for patients who desired organ preservation of the larynx without affecting either DFS or OS. Our data did not reveal clinically relevant differences in the OS between CRT-treated patients and those who underwent surgery. However, we report differences in the DFS rate after five years of follow-up in favor of the surgery-treated group of patients.

Forastiere et al. [27] evaluated three treatments: induction cisplatin plus fluorouracil followed by radiotherapy, radiotherapy with a concurrent administration of cisplatin, and radiotherapy treatment exclusively. They did not find statistically significant differences in the OS between the studied groups at 2 and 5 years of follow-up, but DFS was lower in patients who received radical RT than in those who were treated with induction chemotherapy or concurrent CRT. On the contrary, our findings revealed differences in the estimates of OS and DFS between patients who underwent CRT and those who received RT with radical intent, with a clear inferiority of the results of the latter. We consider it necessary to assess whether the magnitude of the differences observed in OS is due to comorbidities and constitutes an exclusion criterion for chemotherapy administration. On the other hand, DFS should not be influenced for this reason; the radiosensitization power and the preventive effect of distant metastasis of chemotherapy are well established [28], which could explain the differences observed between the CRT and RT groups. 

We did not observe any difference between the OS in patients who underwent surgery or CRT in stages III or IV of LC. However, both techniques were superior to radical radiation therapy in patients with stage III or IV LC. Surgery offers better results than organ preservation therapy with CRT in the case of stage IV LC. Additionally, both surgery and CRT present better survival rates relative to RT therapy alone in the case of stage III LC [29]. 

Our study suffers from certain limitations. The retrospective design of the study made more difficult the control of the potential selection bias. Additionally, we were unable to demonstrate the effects of other comorbidities that share common risk factors with LC. The 10-year follow-up period may not be sufficient to evaluate the impact of each treatment, as deaths due to cardiac and lung pathologies usually occur 15 to 20 years after the initial diagnosis. Finally, the evaluation of chemotherapy was compromised by the high rate of treatment interruptions due to possible related side effects.

## 5. Conclusions

Surgical treatment improves CSS and DFS at five years in patients with initial LC with respect to radiation therapy alone. Furthermore, surgical treatment associated with complementary radiation therapy offers better CSS and DFS in patients with advanced LC. We believe that prospective studies are needed to confirm these results.

## Figures and Tables

**Table 1:** Sociodemographic and clinical baseline characteristics of the study population.

Characteristics	Initial Stages (N = 147)		Advanced Stages (N = 169)	
	N (%)	95%CI	N (%)	95%CI
**Sex**				
Male	135 (91.8)	(87.7; 96.6)	151 (89.3)	(84.4; 94.2)
Female	12 (8.2)	(3.4; 12.9)	18 (10.7)	(5.7; 15.6)
**Tobacco smoking**				
No	19 (12.9)	(7.2; 18.7)	8 (4.7)	(1.2; 8.2)
Yes	128 (87.1)	(81.3; 92.8)	161 (95.3)	(91.8; 98.8)
**Alcohol consumption**				
No	92 (62.6)	(54.4; 70.7)	78 (46.2)	(38.3; 54.0)
Yes	55 (37.4)	(29.2; 45.6)	91 (53.8)	(46.0; 61.6)
**Intravenous drug use**				
No	141 (95.9)	(92.4; 99.4)	158 (93.5)	(89.5; 97.5)
Yes	6 (4.1)	(0.5; 7.6)	11 (6.5)	(2.5; 10.5)
**Tumoral Stage (T)**				
T1	89 (60.5)	(52.3; 68.8)	5 (3.0)	(1.0; 6.8)
T2	58 (39.5)	(31.2; 47.7)	15 (8.9)	(4.3; 13.4)
T3	0 (0.0)	(0.0; 2.5)	92 (54.4)	(46.6; 62.2)
T4	0 (0.0)	(0.0; 2.5)	57 (33.7)	(26.3; 41.1)
**Nodal Metastasis (N)**				
N0	146 (99.3)	(96.3;100.0)	99 (58.6)	(50.8; 66.3)
N1	1 (0.7)	(0.0; 3.7)	16 (9.5)	(4.7; 14.2)
N2	0 (0.0)	(0.0; 2.5)	54 (32.0)	(24.6; 39.3)
N3	0 0.0)	(0.0; 2.5)	0 (0.0)	(0.0; 2.1)
**Tumor location**				
Supraglottic	22 (15.0)	9.9; 21.4	81 (47.9)	40.1; 55.7
Glottic	121 (82.3)	75.5; 87.8	59 (34.9)	27.7; 42.6
Subglottic	3 (2.0)	0.4; 5.8	6 (3.6)	0.4; 6.6
Pyriform sinus Extension	1 (0.7)	0.0; 3.7	19 (11.2)	6.2; 16.3
Transglottic	0 0.0)	0.0; 2.5	4 (2.4)	0.6; 5.9
**Histological grade**				
G1	48 (32.7)	25.5; 40.5	43 (25.4)	19.1; 32.7
G2	75 (51.0)	43.0; 59.0	88 (52.1)	44.3; 59.8
G3	13 (8.8)	5.0; 14.2	29 (17.2)	11.8; 23.7
**Different from squamous cell carcinoma**	11(7.5)	4.0; 12.6	9 (5.3)	1.6; 9.0
Basaloid	2 (1.4)	0.2; 4.8	9 (5.3)	1.6; 9.0
Sarcomatoid	5 (3.4)	1.1; 7.7	0 (0.0)	0.0; 2.1
Verrucous	4 (2.7)	0.7; 6.8	0 (0.0)	0.0; 2.1
**Treatment**				
Surgery	84 (57.1)	48.8; 65.5	111 (65.7)	58.2; 73.1
Radiation Therapy	63 (42.9)	33.8; 50.5	14 (6.3)	3.8; 12.7
Chemoradiotherapy	0 (0.0)	0.0; 3.7	44 (26.0)	19.1; 32.9
**Adjuvant chemotherapy**				
No	145 (98.6)	95.2;100.0	147 (87.0)	81.6; 92.3
Yes	2 (1.4)	0.2; 4.8	22 (13.0)	7.6; 18.4
**Adjuvant radiation therapy**				
No	126 (85.7)	79.7; 91.7	112 (66.3)	58.8; 73.7
Yes	21 (14.3)	8.3; 20.3	57 (33.7)	26.3; 41.1

**Table 2:** Univariate analysis of the risk factors.

	Cause-Specific Survival			Overall Survival			Disease-Free Survival		
Variables	$\hat{HR}$	CI 95%	*p*	$\hat{HR}$	CI 95%	*p*	$\hat{HR}$	CI 95%	*p*
**Older than 70 years**									
- ≤70 - >70	1 1.81	1.07–3.05	0.027	1 2.37	1.57–3.40	0.00005	1 1.53	0.97–2.42	0.067
**Sex**									
- Male - Female	1 0.75	0.30–1.86	0.533	1 0.68	0.33–1.40	0.303	1 1.02	0.54–1.91	0.955
**Tobacco**									
- No - Yes	1 1.79	0.56–5.71	0.323	1 1.54	0.68–3.51	0.302	1 1.11	0.51–2.39	0.790
**Alcohol**									
- No - Yes	1 2.03	1.24–3.33	0.005	1 1.37	0.95–1.97	0.093	1 1.61	1.10–2.37	0.015
**IDU**									
- No - Yes	1 0.90	0.28–2.88	0.863	1 0.52	0.17–1.65	0.270	1 1.34	0.59–3.07	0.484
**Tumor Stage**									
- 1 y 2 - 3 y 4	1 1.98	1.20–3.27	0.008	1 1.53	1.05–2.21	0.026	1 0.86	0.58–1.23	0.430
**Nodal Stage**									
- N0 y N1 - N2 y N3	1 2.26	1.34–3.83	0.002	1 1.66	1.08–2.53	0.020	1 1.71	0.72–1.91	0.526
**Stage**									
- I/II - III/IVa	1 2.89	1.30–6.39	0.009	1 2.01	1.35–2.98	0.001	1 0.95	0.65–1.40	0.813
**Location**									
- Supraglottic - Glottic	1 0.75	0.44–1.29	0.304	1 0.95	0.55–1.63	0.850	1 0.75	0.79–1.79	0.401
**Treatment**									
- Surgery - OPP	1 0.62	0.38–1.02	0.061	1 0.74	0.50–1.90	0.127	1 0.61	0.40–0.91	0.015
**Adyuvant radiation therapy**									
- No - Yes	1 0.84	0.47–1.49	0.543	1 0.69	0.38–1.25	0.223	1 0.46	0.26–0.80	0.006
**Feature post-radiotherapy**									
- No - Yes	1 0.11	0.05–0.28	0.00005	1 0.37	0.20–0.66	0.001	1 0.76	0.34–1.70	0.511
**Local recurrence**									
- No - Yes	1 8.94	4.87–16.41	0.00005	1 1.99	1.37–2.88	0.00005			
**Regional recurrence**									
- No - Yes	1 11.73	7.15–19.23	0.00005	1 4.38	2.89–6.63	0.00005			
**Rescue chemotherapy**									
- No - Yes	1 5.51	3.22–9.43	0.00005	1 2.79	1.71–4.59	0.00005			
**Rescue radiation therapy**									
- No - Yes	1 1.58	0.87–2.85	0.131	1 1.03	0.62–1.70	0.914			

IDU: intravenous drug users and OPP: organ preservation protocol.

**Table 3:** Survival model.

Cause-Specific Survival			
Variables	$\hat{HR}$	CI 95%	*p*
**Stage**			
- I/II - III/IV	1 2.95	1.66–5.22	0.0005
**Treatment**			
- OPP - Surgery	1 0.58	0.35–0.98	0.042
**Overall survival**			
Variables	$\hat{\mathrm{HR}}$	CI 95%	*p*
**Stage**			
- T1 y T2 - T3 y T4	1 3.07	1.57–5.99	0.001
**Age**			
- ≤70 - >70	1 4.23	2.14–8.34	0.00005
**Local recurrence**			
- No - Yes	1 3.48	1.62–7.50	0.001
**Feature post-radiotherapy**			
- No - Yes	1 0.45	0.23–0.87	0.017
**Rescue chemotherapy**			
- No - Yes	1 3.09	1.20–7.98	0.020
**Disease-Free Survival**			
VARIABLES	$\hat{\mathrm{HR}}$	CI 95%	*p*
**Age**			
- ≤70 - >70	1 1.81	1.13–2.90	0.013
**Alcohol intake**			
- No - Yes	11.77	1.20–2.63	0.004
**Adjuvant radiation therapy**			
- No - Yes	1 0.44	0.25–0.78	0.005

## Data Availability

The data sets used and/or analyzed during the current study are available from the corresponding author upon reasonable request.

## References

[1] Bray F., Ferlay J., Soerjomataram I., Siegel R.L., Torre L.A., Jemal A. (2018). Global cancer statistics 2018: GLOBOCAN estimates of incidence and mortality worldwide for 36 cancers in 185 countries. CA Cancer J. Clin..

[2] Zuo J.-J., Tao Z.-Z., Chen C., Hu Z.-W., Xu Y.-X., Zheng A.-Y., Guo Y. (2017). Characteristics of cigarette smoking without alcohol consumption and laryngeal cancer: Overall and time-risk relation. A meta-analysis of observational studies. Eur. Arch. Otorhinolaryngol..

[3] Zhang Z.F., Morgenstern H., Spitz M.R., Tashkin D.P., Yu G.P., Hsu T.C., Schantz S.P. (2000). Environmental tobacco smoking, mutagen sensitivity, and head and neck squamous cell carcinoma. Cancer Epidemiol. Biomark. Prev..

[4] Vineis P., Airoldi L., Veglia F., Olgiati L., Pastorelli R., Autrup H., Dunning A., Garte S., Gormally E., Hainaut P. (2005). Environmental tobacco smoke and risk of respiratory cancer and chronic obstructive pulmonary disease in former smokers and never smokers in the EPIC prospective study. BMJ.

[5] Marziliano A., Teckie S., Diefenbach M.A. (2020). Alcohol-related head and neck cancer: Summary of the literature. Head Neck.

[6] Dhull A.K., Atri R., Dhankhar R., Chauhan A.K., Kaushal V. (2018). Major Risk Factors in Head and Neck Cancer: A Retrospective Analysis of 12-Year Experiences. World J. Oncol..

[7] Leoncini E., Ricciardi W., Cadoni G., Arzani D., Petrelli L., Paludetti G., Brennan P., Luce D., Stucker I., Matsuo K. (2014). Adult height and head and neck cancer: A pooled analysis within the INHANCE Consortium. Eur. J. Epidemiol..

[8] Ferlito A., Silver C.E., Zeitels S.M., Rinaldo A. (2002). Evolution of laryngeal cancer surgery. Acta Otolaryngol..

[9] Wolf G.T., Fisher S.G., Hong W.K., Hillman R., Spaulding M., Laramore G.E., Endicott J.W., McClatchey K., Henderson W.G., Department of Veterans Affairs Laryngeal Cancer Study Group (1991). Induction chemotherapy plus radiation compared with surgery plus radiation in patients with advanced laryngeal cancer. N. Engl. J. Med..

[10] Brierley J.D., Gospodarowicz M.K., Wittekind C. (2017). TNM Classification of Malignant Tumours.

[11] Baird B.J., Sung C.K., Beadle B.M., Divi V. (2018). Treatment of early-stage laryngeal cancer: A comparison of treatment options. Oral Oncol..

[12] Feng Y., Wang B., Wen S. (2011). Laser surgery versus radiotherapy for T1–T2N0 glottic cancer: A meta-analysis. ORL J. Otorhinolaryngol. Relat. Spec..

[13] Chen J.J., Stessin A., Christos P., Wernicke A.G., Nori D., Parashar B. (2015). Differences in survival outcome between stage I and stage II glottic cancer: A SEER-based analysis. Laryngoscope.

[14] Warner L., Chudasama J., Kelly C.G., Loughran S., McKenzie K., Wight R., Dey P. (2014). Radiotherapy versus open surgery versus endolaryngeal surgery (with or without laser) for early laryngeal squamous cell cancer. Cochrane Database Syst. Rev..

[15] Dirix P., Lambrecht M., Nuyts S. (2010). Radiotherapy for laryngeal squamous cell carcinoma: Current standards. Expert Rev. Anticancer Ther..

[16] Lacas B., Carmel A., Landais C., Wong S.J., Licitra L., Tobias J.S., Burtness B., Ghi M.G., Cohen E.E., Grau C. (2021). Meta-analysis of chemotherapy in head and neck cancer (MACH-NC): An update on 107 randomized trials and 19,805 patients, on behalf of MACH-NC Group. Radiother. Oncol..

[17] Spector M.E., Rosko A.J., Swiecicki P.L., Chad Brenner J., Birkeland A.C. (2018). From VA Larynx to the future of chemoselection: Defining the role of induction chemotherapy in larynx cancer. Oral Oncol..

[18] Mesolella M., Iorio B., Buono S., Cimmino M., Motta G. (2021). Supracricoid Partial Laryngectomy: Oncological and Functional Outcomes. Int. Arch. Otorhinolaryngol..

[19] Siegel R.L., Miller K.D., Jemal A. (2016). Cancer statistics, 2016. CA Cancer J. Clin..

[20] Megwalu U.C., Sikora A.G. (2014). Survival outcomes in advanced laryngeal cancer. JAMA Otolaryngol. Head Neck Surg..

[21] Wolf G.T., Bellile E., Eisbruch A., Urba S., Bradford C.R., Peterson L., Prince M.E., Teknos T.N., Chepeha D., Hogikyan N.D. (2017). Survival Rates Using Individualized Bioselection Treatment Methods in Patients with Advanced Laryngeal Cancer. JAMA Otolaryngol. Head Neck Surg..

[22] Sanabria A., Chaves A.L., Kowalski L.P., Wolf G.T., Saba N.F., Forastiere A.A., Beitler J.J., Nibu K.-I., Bradford C.R., Suárez C. (2017). Organ preservation with chemoradiation in advanced laryngeal cancer: The problem of generalizing results from randomized controlled trials. Auris Nasus Larynx.

[23] Bonomi M.R., Blakaj A., Blakaj D. (2018). Organ preservation for advanced larynx cancer: A review of chemotherapy and radiation combination strategies. Oral Oncol..

[24] Patel K.B., Nichols A.C., Fung K., Yoo J., MacNeil S.D. (2018). Treatment of early stage Supraglottic squamous cell carcinoma: Meta-analysis comparing primary surgery versus primary radiotherapy. J. Otolaryngol. Head Neck Surg..

[25] Mo H.-L., Li J., Yang X., Zhang F., Xiong J.-W., Yang Z.-L., Tan J., Li B. (2017). Transoral laser microsurgery versus radiotherapy for T1 glottic carcinoma: A systematic review and meta-analysis. Lasers Med. Sci..

[26] De Santis R.J., Poon I., Lee J., Karam I., Enepekides D.J., Higgins K.M. (2016). Comparison of survival between radiation therapy and trans-oral laser microsurgery for early glottic cancer patients; a retrospective cohort study. J. Otolaryngol. Head Neck Surg..

[27] Forastiere A.A., Goepfert H., Maor M., Pajak T.F., Weber R., Morrison W., Glisson B., Trotti A., Ridge J.A., Chao C. (2003). Concurrent Chemotherapy and Radiotherapy for Organ Preservation in Advanced Laryngeal Cancer. N. Engl. J. Med..

[28] Haigentz M., Silver C.E., Hartl D.M., Takes R.P., Rodrigo J.P., Robbins K.T., Rinaldo A., Ferlito A. (2010). Chemotherapy regimens and treatment protocols for laryngeal cancer. Expert Opin. Pharmacother..

[29] Gourin C.G., Conger B.T., Sheils W.C., Bilodeau P.A., Coleman T.A., Porubsky E.S. (2009). The effect of treatment on survival in patients with advanced laryngeal carcinoma. Laryngoscope.

